# Effectiveness of a Telecare-Based Intervention Program in Supporting Informal Caregivers of Community-Dwelling Older Adults in Reducing Stress Levels: Randomized Controlled Trial

**DOI:** 10.2196/70791

**Published:** 2026-04-07

**Authors:** Arkers Kwan Ching Wong, Jonathan Bayuo, Nga Ping Ng, Matthew Yau, Ka Kit Simon Yu, Rose Sin Yi Lin, Jing Jing Su, Vivian Hui, Jed Montayre

**Affiliations:** 1 School of Nursing, Faculty of Health and Social Science, The Hong Kong Polytechnic University, Room 502, Block GH, 1 Cheong Wan Road, Hung Hom, Hong Kong, China (Hong Kong), 852 34003805; 2 School of Nursing and Midwifery, University of Southern Queensland, Brisbane, Australia; 3 The Salvation Army, Hong Kong, China (Hong Kong); 4 School of Nursing, University of Rochester, Rochester, NY, United States; 5 School of Nursing, Tung Wah College, Homantin, China (Hong Kong)

**Keywords:** older adults, informal caregivers, caregiving, care, stress, telecare, community

## Abstract

**Background:**

Informal caregivers are essential in supporting community-dwelling older adults, especially as global populations age. However, caregiving responsibilities often result in high stress, depressive symptoms, and diminished quality of life. Traditional support services are fragmented, and many programs overlook caregivers’ emotional and informational needs. Telecare—the remote delivery of health and social care via digital technologies—offers a promising approach to provide accessible, personalized, and timely support.

**Objective:**

This study evaluated the effectiveness of a telecare-based intervention program, developed by a health-social partnership team, in improving psychological well-being and caregiving outcomes among informal caregivers of community-dwelling older adults. We hypothesized that caregivers in the intervention group would show greater improvements in stress, self-efficacy, depression, quality of life, and caregiving burden compared to those receiving usual care.

**Methods:**

A single-blind randomized controlled trial was conducted from January to December 2023, with 75 informal caregivers providing care to older adults (aged ≥60 y) for at least 4 hours per week. Participants were randomized to an intervention group (n=38, 51%) or a control group (n=37, 49%). The 3-month intervention included (1) biweekly online nurse case management supported by a multidisciplinary health-social team, (2) personalized WhatsApp videos on caregiving skills, and (3) a password-protected caregiver website offering resources and peer discussion. The control group attended 6 in-person educational sessions at a community center. Outcomes such as stress, self-efficacy, depression, quality of life, and caregiving burden were measured at baseline and postintervention.

**Results:**

Participants had a mean age of 65.5 (SD 9.617) years, and 81.3% (61/74) were female. Baseline characteristics were similar between groups. Compared to controls, the intervention group showed significantly higher self-efficacy (*t*=1.98; *P*=.04) and lower depression (*t*=−2.24; *P*=.03) at follow-up. Within-group improvements in the intervention group were also observed for stress, depression, self-efficacy, mental quality of life, and caregiving burden. No significant between-group differences were found for stress, quality of life, or caregiving burden. No significant within-group improvements were observed in the control group.

**Conclusions:**

This study provides novel evidence that a digitally delivered, nurse-led telecare program—integrating health and social services—can improve psychological outcomes among informal caregivers. These findings have practical implications for health systems seeking scalable, low-barrier solutions to support caregivers. Telecare models may be a valuable complement to traditional services, enhancing caregiver resilience and promoting aging in place while alleviating long-term strain on institutional care systems.

## Introduction

### Background

With declining fertility and increasing life expectancy, global populations are aging rapidly. In Hong Kong, the proportion of older adults is projected to rise from 17.9% in 2018 to 31.9% by 2038, placing increasing pressure on health care and welfare systems [1,2]. Most older adults prefer aging in place over institutional care, even when frail, making informal caregivers central to community-based elderly care [3].

These caregivers assist with daily tasks, medical appointments, and decision-making, often acting as crucial advocates for their loved ones [4,5]. While their contributions help reduce health care use and improve outcomes for older adults, caregiving is physically and emotionally demanding. Many caregivers face limited access to support, inadequate training, and social isolation, leading to feelings of guilt, fatigue, and mental distress. Studies show that more than 60% of caregivers experience high caregiving burden, 55% report depressive symptoms, and 40% face poor family functioning [6-8].

Despite their vital role, informal caregivers often receive far less support than care recipients. In Hong Kong, the lack of an integrated health and social care system contributes to fragmented services, making it difficult for caregivers to seek timely and coordinated assistance [9]. A local study in 2022 found that many caregivers expressed a strong need for a centralized platform offering information, caregiving resources, and service referrals [10,11].

Advances in digital health offer promising solutions. Telecare, which leverages information and communication technologies, enables remote monitoring and timely support from health care professionals [12,13]. It is especially useful for working caregivers, offering both practical information and emotional support without the constraints of time and location [3,14].

Several systematic reviews have demonstrated that telehealth or telecare interventions—often delivered via phone or videoconferencing—can moderately reduce caregiver stress, burden, and depressive symptoms, particularly among those caring for individuals with chronic conditions or dementia [14,15]. However, most studies have focused on specific clinical populations and lacked integration with broader community-based health and social services [14]. Few programs have incorporated real-time digital case management, asynchronous tailored messaging, and interactive web-based peer support in a comprehensive model. Furthermore, although some telecare interventions have been tested among older adults with multimorbidity and young populations [16], there remains a significant gap in research targeting informal caregivers of community-dwelling older adults with diverse needs. Addressing these gaps, this study evaluates the effectiveness of a multicomponent telecare program, co-developed by a health-social partnership team, in improving caregivers’ psychological well-being and caregiving outcomes. We hypothesized that participants in the intervention group would demonstrate greater reductions in stress and depression and greater improvements in self-efficacy, quality of life, and caregiving burden compared to those receiving usual care.

### Conceptual Framework

The design of this program was informed by Lazarus and Folkman’s [17] transactional theory of stress and coping and the COPE (Creativity, Optimism, Planning, and Expert Information) model. In line with the transactional theory, the intervention aimed to reduce caregiver stress by enhancing their appraisal and coping capacity. Caregivers were supported to adopt problem-focused strategies (eg, goal setting and seeking solutions) and emotion-focused strategies (eg, stress management and emotional support) to manage caregiving challenges [18].

The COPE model provided a practical framework for empowering caregivers through the intervention components. For instance, creativity was encouraged through guided problem-solving discussions; optimism was fostered by reframing challenges and sharing success stories; planning was embedded in structured goal-setting and action plans; and expert information was delivered via health-social professionals and curated digital content [19]. Together, these models guided the development of a multicomponent program that aimed to enhance caregivers’ psychological well-being and self-efficacy in managing care responsibilities.

## Methods

### Overview

The details can be found in the published protocol [20]. The reporting of the trial adhered to the CONSORT (CONsolidated Standards Of Reporting Trials) 2025 checklist (Checklist 1).

### Study Design and Setting

This is a single-blinded, 2-armed randomized controlled trial. The study was conducted in collaboration with one of the largest nongovernmental organizations in the world, which has more than 1.7 million older members.

### Participants, Recruitment Strategy, and Randomization

The criteria for the inclusion of participants were as follows: (1) being aged ≥18 years; (2) having no cognitive impairment; (3) being able to understand and communicate in Cantonese; (4) providing care for older adults aged ≥60 years for a minimum of 4 hours per week over a period of at least 3 months; (5) being a current smartphone user; (6) having knowledge of how to access the internet; (7) committing to attend biweekly online meetings with the program providers, lasting 15 to 30 minutes, over a period of 3 months; and (8) being willing to receive individual-specific video messages covering caregiving skills via WhatsApp (Meta Platforms Inc.). Exclusion criteria included (1) being an alcoholic or using psychiatric drugs, (2) being illiterate (inability to read and write), (3) having a diagnosis of chronic mental problems, and (4) having previously participated in health or social programs offered by other organizations.

Participants were recruited between January 2023 and September 2024. The intervention was delivered from January 2023 to December 2024. In community centers, a trained research assistant assessed and recruited eligible participants by sharing details of the study with them and inviting them to join. Verbal consent was obtained from those who agreed to join the program. Following the collection of baseline data, the research assistant contacted the principal investigator of the research team to proceed with randomization. The principal investigator used a software program called “Research Randomizer” to generate numbers, which were placed in sealed envelopes. Each envelope corresponded to either the intervention group (“1”) or the control group (“2”). The envelopes were opened sequentially upon receiving a call from the research assistant. In this study, the research assistant was blinded to group assignments, whereas the principal investigator and health care providers were not blinded.

### Intervention Group

#### Overview

The participants in the telecare-based intervention group received a 3-month program consisting of three intervention components: (1) online nurse case management supported by a health-social partnership team, (2) individual-specific video messages covering caregiving skills via WhatsApp, and (3) an online information center and discussion forum accessed via a password-protected, newly developed caregiver website.

#### Online Nurse Case Management Supported by a Health-Social Partnership Team

Online case management was facilitated by a registered nurse who played a leading role, along with a team of health care professionals from various disciplines, including physiotherapists, Chinese medicine practitioners, and social workers. The registered nurse conducted individual Zoom meetings with each participant in the intervention group every 2 weeks, for a total of 6 Zoom meetings per participant throughout the intervention period. During the initial meeting, the nurse encouraged caregivers to share their current situation and explored any needs or difficulties they were facing, using the Omaha system. The Omaha system comprises the Problem Classification Scheme, the Problem Rating Scale for Outcomes, and the Intervention Scheme [20,21]. This structured approach assisted the nurse in identifying the health needs of the caregivers, setting intervention goals, and evaluating the effectiveness of the intervention. Subsequently, personalized care plans were developed for each participant. The nurse provided recommendations and consulted other health care professionals from relevant disciplines as needed. Follow-up meetings involved ongoing communication with caregivers to discuss any updated situations and provide tailored physical, social, and emotional support based on their specific needs. The nurses offered additional resources and made referrals to the health-social partnership team whenever necessary. This approach leveraged technology to facilitate the provision of coordinated care tailored to the unique needs of each individual. It involved coordinating the efforts of health care professionals to ensure that the clients’ requirements were effectively met.

#### Individual-Specific Video Messages Covering Caregiving Skills Via WhatsApp

With the widespread popularity of WhatsApp, the registered nurse used this platform to offer additional support to the caregivers. The nurse sent individualized messages or videos weekly to the participants through WhatsApp, focusing on their needs as identified from Zoom meetings. The information that was shared encompassed caregiving knowledge and skills, self-care recommendations, health information, and tips for managing emotions. Using this convenient communication tool, the nurse ensured the timely delivery of information while also providing an opportunity for participants to engage in asynchronous communication with the nurse. In the event that participants had any questions or concerns, they could easily respond to the nurse’s messages through WhatsApp, allowing the nurse to provide prompt responses and support.

#### Online Information Center and Discussion Forum Via a Password-Protected, Newly Developed Caregiver Website

A new website was created specifically for informal caregivers, consisting of two components: an information center and a discussion forum. At the beginning of the intervention period, each participant was randomly assigned a preset username and password to log into the website. The usernames were kept anonymous to encourage participants to take part without hesitation, and the passwords were securely protected.

The research assistant played a role in maintaining the website by posting 2 to 3 pieces of useful information in the information center on a weekly basis for caregivers to read. Caregivers were able to comment on these posts and raise questions related to caregiving. Members of the health-social partnership team promptly replied to these questions, providing valuable insights and guidance.

Regarding the discussion forum, participants had the freedom to initiate any caregiving-related questions or discuss the challenges they were facing at any time. The members of the health-social team were available to respond to these questions, and participants were also encouraged to interact with each other, sharing their perspectives and providing suggestions. To foster engagement, the research assistant occasionally initiated new discussion topics, encouraging participants to share their own experiences.

The website served as more than just a source of knowledge and information for caregivers; it also provided them with a platform to express their feelings, receive encouragement, and connect with other caregivers who shared similar experiences.

### Control Group

In addition to the telecare-based intervention group, the control group also received the usual services from community centers. The participants in the control group attended 6 face-to-face educational talks during the intervention period, which took place at the community center. These talks were conducted by members of the health-social partnership team, including a registered nurse, a Chinese medicine practitioner, a physiotherapist, and a mental health nurse.

Each talk covered 1 or 2 specific topics and lasted approximately 2 hours. The health care professionals provided informal caregivers with knowledge and live demonstrations related to self-care and elderly care. The topics addressed during the talks included the management of chronic diseases, acupressure techniques, eye and oral care for older adults, pain management strategies, lifting and transfer techniques, fall prevention measures, swallowing issues, and the management of insomnia. During the questions and answers session of each talk, participants had the opportunity to ask questions and seek clarification on the topics that had been discussed. Upon completion of the follow-up data collection, participants in the control group were offered access to the online caregiver platform and its educational resources to ensure equitable support and knowledge dissemination.

### Data Collection

The data were collected at two time points: at baseline (T1) and at the follow-up after the interventions (T2). Data collection for the study involved the use of Qualtrics, a survey platform, where participants were provided with a link to complete the questionnaires themselves. However, for participants who encountered difficulties in filling out the questionnaires online, alternative methods were used. In such cases, data collection took place face-to-face with the assistance of the research assistant and staff from the nongovernmental organizations. They provided support and guidance to the participants, ensuring that the questionnaires were completed accurately and comprehensively. This approach allowed for flexibility in data collection, accommodating participants who may have experienced challenges with completing the online survey.

### Outcome Measures

#### Primary Outcome

##### The 14-Item Chinese Version of the Perceived Stress Scale

The primary outcome of the study was the stress level of the caregivers. The 14-item Chinese version of the Perceived Stress Scale was used to evaluate the participants’ perception of stress and the degree to which they perceived their lives to be uncontrollable [22]. All items are rated on a 5-point Likert scale, ranging from 0 (never) to 4 (very often), and 7 of them (4, 5, 6, 7, 9, 10, and 13) are reverse-scored, with higher scores representing lesser perceived stress. The overall Cronbach α of the Chinese version of the Perceived Stress Scale was 0.906, indicating good reliability [23].

### Secondary Outcomes

The secondary outcomes included self-efficacy, depression, quality of life, and caregiving burden of the caregivers.

#### Self-Efficacy—The Chinese Version of the General Self-Efficacy Scale

The self-efficacy of the individuals was assessed using the Chinese version of the General Self-Efficacy Scale. It is a scale that is widely used to assess an individual’s belief in his or her own capacity to cope with stressful situations or difficulties [24,25]. It contains 10 items, and scores for each item range from 1 (not at all true) to 4 (exactly true). The higher the total score, the greater the individual’s self-efficacy. The reliability of the Chinese version of the General Self-Efficacy Scale is good, with a Cronbach α of >0.91, indicating internal consistency [26]. The scale has been shown to have good criterion validity and good psychometric properties [27].

#### Depression—The Chinese Version of the Center for Epidemiologic Studies Depression Scale

The Chinese version of the Center for Epidemiologic Studies Depression Scale is a widely used self-reported questionnaire consisting of 20 items that assess depression symptomatology. It is designed to measure the frequency and severity of depressive symptoms experienced by individuals. The scale has been widely used in research and clinical settings to assess symptoms of depression in Chinese-speaking populations [28-30]. The Chinese version of the Center for Epidemiologic Studies Depression Scale has been validated, with a Cronbach α and intraclass correlation coefficient of the total scale and subscales ranging from 0.82 to 0.88 and from 0.73 to 0.81, respectively [31].

#### Quality of Life—The Chinese Short Form-12 Health Survey Version 2

The Chinese Short Form-12 Health Survey version 2 is a measure used to assess health-related quality of life. It evaluates 8 domains, namely physical functioning, role physical, bodily pain, general health, vitality, social functioning, role emotional, and mental health [32]. It consists of 12 items divided into a physical component summary score and a mental component summary score [33]. Higher scores indicate that an individual has a better health-related quality of life. The reliability of the Chinese Short Form-12 Health Survey version 2 was good, with a Cronbach α of 0.81 [34].

#### Caregiving Burden—The Short Form of the Zarit Burden Interview

The Chinese version of the Zarit Burden Interview is a 22-item scale widely used to assess the level of caregiving burden of informal caregivers [35]. The response to each item is rated on a 5-point scale, ranging from 0 (never) to 4 (always). The total score ranges from 0 to 88, with higher scores indicating a greater caregiving burden. The Chinese version of the short form of the Zarit Burden Interview demonstrates very good internal consistency, with a Cronbach α of 0.875 [36,37].

#### Background Demographic Data

The background demographic data were collected at baseline (T1). They included age, gender, marital status, educational level, employment, economic status, caregiving experience, time of providing care to the older adult per week, relationship with the care recipient, and experience using a smartphone.

### Sample Size

A 2-tailed alpha of .05, a power of 80% (a beta error probability of .2), and an effect size of 0.403 were used to determine the size of the sample. This effect size was based on a recent telephone-based mobile health program that assessed the same primary outcome measure as in this study, namely perceived stress level [38]. On the basis of these considerations, a sample size of 34 individuals per group was necessary.

Considering an attrition rate of 10% to 15% reported in a previous program [38], a dropout rate of 15% was assumed for this study. Taking into account this dropout rate, the total sample size required was 80 participants, with 40 participants allocated to each group.

### Statistical Analysis

The statistical analysis was conducted using the Statistical Package for the Social Sciences (version 29.0). To compare the demographic data of the intervention and control groups at baseline, the Mann-Whitney *U* test, *t* tests, and chi-square tests were used. Normality was tested using frequency distribution histograms, and all data were found to conform to a normal distribution. Therefore, independent *t* tests (two-tailed) were used to analyze the mean differences in primary and secondary outcomes between the two groups, and paired *t* tests (two-tailed) were used to compare within-group changes before and after the intervention. A primary intention-to-treat analysis was performed. Missing outcome data for participants lost to follow-up were addressed using multiple imputation based on the assumption of missing completely at random. A significant result was determined when the *P* value (level of significance) was .04 for a 2-tailed test, indicating a statistically significant difference.

### Ethical Considerations

This study was approved by the Human Subjects Ethics Sub-Committee of the Hong Kong Polytechnic University (reference number HSEARS20210907001). All participants were provided with detailed information about the study’s purpose, procedures, risks, and benefits. Written informed consent was obtained from all participants before data collection, and they were informed of their right to withdraw at any time without consequences. All collected data were anonymized, and identifying information was removed before analysis to ensure confidentiality. Secure storage procedures were implemented to safeguard digital and paper records. No personal identifiers were used in reporting results. Participants received a supermarket coupon valued at HK $50 (approximately US $6.50) as a token of appreciation for their time and effort.

## Results

### Baseline Characteristics

**Figure 1.**
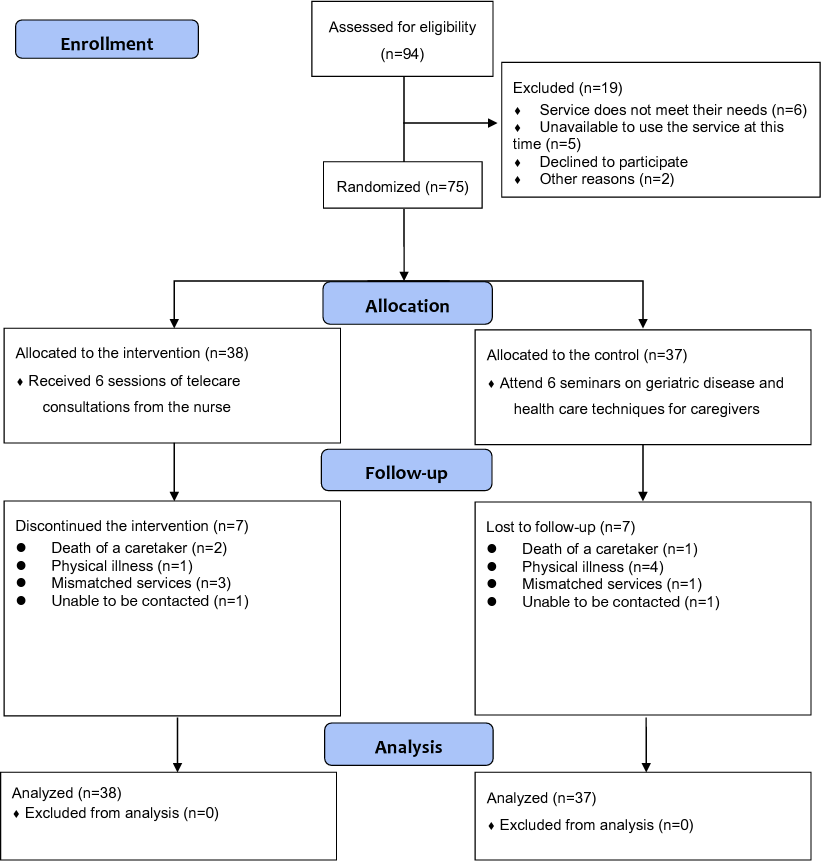
CONSORT (Consolidated Standards Of Reporting Trials) flow diagram.

A total of 94 participants were assessed for eligibility. Of these, 75 (79.8%) individuals (n=38, 51%, in the intervention group; and n=37, 49%, in the control group) were eventually recruited and randomized for the study. Seven participants from both groups dropped out for various reasons, such as the death of a caretaker, physical illness, mismatched services, or they were unable to be contacted (Figure 1). Among the 75 participants, 14 (18.7%) were male and 61 (81.3%) were female, with a mean age of 65.5 years. Approximately 62.7% (47/75) were retired, and 52.0% (39/75) were caregivers for their parents. A significant proportion (44/75, 58.6%) had been taking care of older adults for 5 years or longer. All participants were experienced in using smartphones. There were no statistically significant differences in participant characteristics or outcome measures between the groups at baseline. Table 1 presents the demographic characteristics of the participants at baseline.

**Table 1.** Baseline characteristics of both the intervention and control groups.

Characteristics	Total (n=75)	Intervention group (n=38)	Control group (n=37)	*P* value
Gender, n (%)				.59
Female	61 (81.3)	30 (78.9)	31 (83.8)	
Male	14 (18.7)	8 (21.1)	6 (16.2)	
Age (y)				.22
Mean (SD)	65.5 (9.617)	64.2 (9.519)	66.7 (9.686)	
Median (range)	67 (44‐84)	65.5 (44-81)	70 (46‐84)	
Age range (y), n (%)				.22
40-50	6 (8.0)	4 (10.5)	2 (5.4)	
51-60	17 (22.7)	9 (23.7)	8 (21.6)	
61-70	29 (38.7)	16 (42.1)	13 (35.1)	
71-80	20 (26.7)	8 (21.1)	12 (32.4)	
81-84	3 (4.0)	1 (2.6)	2 (5.4)	
Marital status, n (%)				.14
Not married	53 (70.7)	23 (60.5)	30 (81.1)	
Married	19 (25.3)	13 (34.2)	6 (16.2)	
Divorced	3 (4.0)	2 (5.3)	1 (2.7)	
Education level, n (%)				.10
Primary	9 (12.0)	4 (10.5)	5 (13.5)	
Secondary	42 (56.0)	19 (50.0)	23 (62.2)	
Tertiary or above	22 (29.3)	15 (39.5)	7 (18.9)	
Others	2 (2.7)	0 (0.0)	2 (5.4)	
How many hours do you spend caring for your care recipient? (on a weekly basis) (h), n (%)				.21
1‐10	14 (18.7)	5 (13.2)	9 (24.3)	
11‐20	7 (9.3)	5 (13.2)	2 (5.4)	
21‐30	13 (17.3)	6 (15.8)	7 (18.9)	
31‐40	5 (6.7)	3 (7.9)	2 (5.4)	
41‐50	2 (2.7)	2 (5.3)	0 (0.0)	
51‐60	7 (9.3)	3 (7.9)	4 (10.8)	
61‐70	3 (4.0)	3 (7.9)	0 (0.0)	
71‐80	2 (2.7)	1 (2.6)	1 (2.7)	
81‐90	1 (1.3)	0 (0.0)	1 (2.7)	
91‐100	0 (0.0)	0 (0.0)	0 (0.0)	
101‐110	1 (1.3)	0 (0.0)	1 (2.7)	
111‐120	2 (2.7)	2 (5.3)	0 (0.0)	
121‐130	2 (2.7)	1 (2.6)	1 (2.7)	
131‐140	0 (0.0)	0 (0.0)	0 (0.0)	
141‐150	1 (1.3)	1 (2.6)	0 (0.0)	
151‐160	1 (1.3)	0 (0.0)	1 (2.7)	
161‐170	14 (18.7)	6 (15.8)	8 (21.6)	
Relationship with the care recipient, n (%)				.10
Spouse	33 (44.0)	13 (34.2)	20 (54.1)	
Parents	39 (52.0)	22 (57.9)	17 (45.9)	
Sibling	1 (1.3)	1 (2.6)	0 (0.0)	
Others	2 (2.7)	2 (5.3)	0 (0.0)	
Experience of taking care of the care recipient, n (%)				.20
<6 mo	6 (8.0)	2 (5.3)	4 (10.8)	
6 mo to 1 y	6 (8.0)	5 (13.2)	1 (2.7)	
2-5 y	19 (25.3)	11 (28.9)	8 (21.6)	
5-10 y	31 (41.3)	16 (42.1)	15 (40.5)	
>10 y	13 (17.3)	4 (10.5)	9 (24.3)	
Occupation, n (%)				.06
Full time	11 (14.7)	10 (26.3)	1 (2.7)	
Part time	1 (1.3)	0 (0.0)	1 (2.7)	
Unemployed	1 (1.3)	0 (0.0)	1 (2.7)	
Retired	47 (62.7)	23 (60.5)	24 (64.9)	
No intention to find a job	15 (20.0)	5 (13.2)	10 (27.0)	
Living (more than one option), n (%)				
Alone	5 (5.6)	3 (7.1)	3 (6.1)	.62
With parents	12 (13.3)	7 (16.7)	5 (10.2)	.56
With spouse	41 (45.5)	16 (38.1)	25 (51.0)	.03
With children	15 (16.7)	6 (14.3)	9 (18.4)	.36
With domestic helper	12 (13.3)	7 (16.7)	5 (10.2)	.53
With sibling	5 (5.6)	3 (7.1)	2 (4.1)	1.00
Experience of using a smartphone (y), n (%)				.11
3‐6	18 (24.0)	10 (26.3)	8 (21.6)	
7‐10	40 (53.3)	18 (47.4)	22 (59.5)	
11‐14	5 (6.7)	2 (5.3)	3 (8.1)	
15‐18	7 (9.3)	4 (10.5)	3 (8.1)	
>18	5 (6.7)	4 (10.5)	1 (2.7)	.

### Perceived Stress Level

There were no statistically significant differences in the means between the 2 groups at T2. However, a statistically significant within-group difference was found in the intervention group, where the perceived stress level of the participants was lower at postintervention than preintervention (*t*=−2.21; *P*=.04). No within-group difference was observed in the control group.

### Self-Efficacy

The intervention group had statistically significant higher self-efficacy scores (*t*=1.98; *P*=.04) when compared to the control group. There was also a within-group effect on self-efficacy level in the intervention group (*t*=−2.28; *P*=.03).

### Depression

The participants in the intervention group had statistically lower depression scores than those in the control group (*t*=−2.24; *P*=.03). The intervention group showed a significant within-group effect (*t*=2.98; *P*=.009), whereas no significant within-group effect was observed in the control group.

### Quality of Life

There were no statistically significant within-group effects in either the intervention group or the control group from T1 to T2 in the physical components of quality of life. A statistically significant within-group difference was observed in the intervention group from T1 to T2 in the mental components of quality of life. There was no between-group difference at T2 in either the physical or mental components of quality of life.

### Caregiving Burden

Similar to perceived stress levels, there was no statistically significant between-group difference at T2 for caregiving burden. However, a statistically significant within-group difference was found in the intervention group from T1 to T2 (*t*=2.89; *P*=.009), where participants had a lower caregiving burden postintervention than preintervention.

Comparisons within and between groups in the pretest and posttest mean scores for all primary and secondary outcomes can be found in Table 2.

**Table 2.** Within- and between-group comparisons of the pretest and posttest mean scores for primary and secondary outcomes.

Variables	Intervention group (n=38), mean (SD)	Control group (n=37), mean (SD)	*t*	*P* value
Perceived stress level				
Pretest	30.35 (7.08)	31.47 (8.10)	−0.47^a^	.64
Posttest	32.58 (8.01)	33.47 (6.84)	−0.46^a^	.64
*t*	−2.21^b^	−1.12^b^	N/A^c^	N/A
*P* value	.04^d^	.27	N/A	N/A
Self-efficacy				
Pretest	24.00 (5.01)	24.93 (4.86)	−0.77^a^	.44
Posttest	26.90 (6.53)	23.27 (6.50)	1.98^a^	.04^d^
*t*	−2.28^b^	0.90^b^	N/A	N/A
*P* value	.03^d^	.84	N/A	N/A
Depression				
Pretest	16.16 (7.93)	15.03 (12.57)	.11^a^	.91
Posttest	10.07 (9.05)	14.16 (8.66)	−2.24^a^	.03^d^
*t*	2.98^b^	1.11^b^	N/A	N/A
*P* value	.009^d^	.29	N/A	N/A
Quality of life				
Physical component				
Pretest	43.46 (8.86)	42.11 (9.15)	.81^a^	.42
Post-test	44.77 (7.30)	42.45 (9.20)	1.09^a^	.28
t	−0.81^b^	−0.30^b^	N/A	N/A
*P* value	.42	.77	N/A	N/A
Mental component				
Pretest	43.95 (11.51)	47.39 (12.61)	−0.74^a^	.32
Posttest	49.48 (8.20)	48.11 (9.37)	.61^a^	.55
*t*	−1.99^b^	−0.96^b^	N/A	N/A
*P* value	.04^d^	.58	N/A	N/A
Caregiving burden				
Pretest	40.90 (16.47)	32.20 (15.27)	1.80^a^	.075
Posttest	30.83 (18.40)	35.32 (16.99)	−.99^a^	.33
*t*	2.89^b^	−0.89^b^	N/A	N/A
*P* value	.009^d^	.60	N/A	N/A

a Independent *t* test.

b Paired *t* test.

c N/A: not applicable.

d *P*<.05; statistical tests used: chi-square test/Kruskal-Wallis test.

## Discussion

### Principal Findings

As populations increasingly age, the role of informal caregivers remains critical in promoting aging in place and ensuring continuity of care. This randomized controlled trial evaluated the effectiveness of a theory-informed, telecare-based intervention in improving caregiver outcomes. The findings demonstrate that caregivers who received the intervention reported significantly higher self-efficacy and lower levels of depression compared to those receiving usual care. Additionally, significant within-group improvements were observed in stress levels, mental quality of life, and caregiving burden in the intervention group, although not reflected in between-group comparisons.

The findings of this randomized controlled trial provide evidence that a telecare-based intervention, grounded in psychological and behavioral theories, can significantly improve depression and self-efficacy among informal caregivers of older adults. These findings align with prior studies demonstrating that accessible, technology-enabled interventions can offer caregivers both emotional support and practical guidance, leading to enhanced psychological resilience and self-management skills [39,40]. Notably, the individualized approach in this intervention—through nurse-led case management and tailored video content—may have allowed caregivers to address their unique caregiving challenges, which supports findings from prior personalized eHealth models [41].

While between-group differences were not observed for perceived stress and caregiving burden, significant within-group improvements in these domains suggest that the intervention had a positive psychosocial effect. This reinforces the importance of early and sustained caregiver support, particularly in settings where community resources are fragmented. Given the rising demand for informal care and global efforts to enable aging in place, these results contribute to a growing body of literature supporting the integration of digital care into community-based services.

The observed reduction in depression among participants may also be attributed to the intervention’s multidimensional support system. Caregivers had multiple channels for engagement, including structured video consultations, asynchronous communication via WhatsApp, and peer interaction on a dedicated web platform. This combination not only improved knowledge and problem-solving capabilities but may also have addressed the social isolation frequently reported by caregivers [42]. The inclusion of personalized content likely enhanced relevance and motivation, 2 factors known to influence behavior change in caregiver populations [43].

The lack of significant improvement in the physical components of quality of life may be attributed to the short duration of the intervention, as changes in physical functioning often require longer follow-up or more intensive caregiver support services. Additionally, variations in care recipients’ health status and caregiving intensity could have influenced these outcomes. Future studies should consider stratifying analyses by caregiving complexity and incorporating longer-term follow-up, cost-effectiveness analysis, and implementation evaluation to better understand differential effects and inform wider adoption.

### Limitations

Several limitations should be noted. First, this trial was prospectively powered for the primary outcome and not powered for subgroup analyses or for detecting smaller effects on secondary outcomes; these estimates should therefore be interpreted with caution. Second, the intervention could not be blinded to participants or providers, potentially introducing response bias. Third, some participants encountered technical difficulties with WhatsApp and the online forum, suggesting that digital literacy and access may affect scalability. Fourth, the duration and content of nurse case management sessions were not formally tracked, limiting fidelity assessment. Fifth, only short-term effects were measured; long-term follow-up is needed to assess sustained impact.

Additionally, we did not collect data on care recipients’ functional status, digital literacy, or the caregivers’ relationship to the care recipient and caregiving network size. These contextual factors may influence intervention effectiveness and should be considered in future research to enable subgroup analyses and more tailored support.

### Conclusions

This study demonstrates that integrating health-social partnership teams with telecare services can significantly improve caregivers’ psychological outcomes. Such community-based interventions offer accessible, tailored support to informal caregivers, helping to reduce emotional burden and promote mental well-being—both of which are essential to sustaining long-term caregiving capacity and ensuring quality of care.

## Supplementary material

10.2196/70791 Checklist 1 CONSORT checklist.

## Abbreviations

CONSORT: CONsolidated Standards Of Reporting Trials
COPE: Creativity, Optimism, Planning, and Expert Information
